# Simvastatin-Loaded Nanoniosome Protects H9c2 Cells from Oxygen-Glucose Deprivation/ Reperfusion Injury by Downregulating Inflammation

**DOI:** 10.61186/ibj.3994

**Published:** 2023-09-30

**Authors:** Maryam Naseroleslami, Mahdieh Mehrab Mohseni

**Affiliations:** Department of Cellular and Molecular Biology, Faculty of Advanced Science and Technology, ‎Tehran Medical Sciences, Islamic Azad University, Tehran, Iran

**Keywords:** Necroptosis, Reperfusion injury, Simvastatin

## Abstract

**Background:**

Simvastatin has anti-inflammatory and antioxidant properties against cardiac I/RI. However, it suffers from low bioavailability and a short half-life. Nanoniosomes are novel drug delivery systems that may increase SIM effectiveness. The present research evaluates the impact of SIM-loaded nanoniosomes on the OGD/R injury model of H9c2 cells.

**Methods:**

Cells were seeded based on five groups: (1) control; (2) OGD/R; (3) OGD/R receiving SIM; (4) OGD/R receiving nanoniosomes; and (5) OGD/R receiving SIMloaded nanoniosomes. OGD/R injury of the H9c2 cells was treated with SIM or SIMloaded nanoniosomes. Cell viability, two inflammatory factors, necroptosis factors, along with *HMGB1* and *Nrf2* gene expressions were assessed.

**Results:**

The cells treated with SIMloaded nanoniosomes showed a significant elevation in the cell viability and a reduction in *HMGB1*, *Nrf2*, *TNF-α*, *IL-1β*, *RIPK1*, and *ROCK1* expression levels compared to the OGD/R and SIM groups.

**Conclusion:**

Based on our findings, nanoniosomes could safely serve as a drug delivery system to counterbalance the disadvantages of SIM, resulting in improved aqueous solubility and stability.

## INTRODUCTION

Myocardial infarction is considered a clinical manifestation of I/RI, which heart failure is its fatal consequence all over the world^[^^1^^]^. Owing to the intricate nature of I/RI disease, its exact pathophysiology has not been fully understood. However, it has been reported that inhibiting cell death pathways could potentially reduce nearly half of the ultimate infarct size^[^^2^^,^^3^^]^. The underlying patho-physiology of I/RI includes oxidative stress (due to excessive production of ROS^[^^4^^]^), abnormal increase in calcium, mitochondrial malfunction, and an inflammatory response that eventually triggers cell death signaling like necrosis, apoptosis, and necroptosis^[^^2^^,^^5^^,^^6^^]^. Hence, finding appropriate treatment for I/RI disease, as one of the most important medical problems, would be a lifesaver. 

SIM, as a second-generation HMG-CoA reductase inhibitor, is primarily prescribed for decreasing cholesterol levels. Moreover, it can possess anti-inflammatory, antioxidant, antiapoptotic, and antifibrotic properties against cardiac I/RI, independent of its cholesterol-lowering role^[^^7^^,^^8^^]^. However, the oral administration of this lipophilic statin has disadvantages such as low bioavailability (less than 5%) and a short half-life (1-2 h) due to the degradation within the gastrointestinal tract and hepatic first-pass metabolism that limits its efficacy and potency^[^^9^^]^. Although there is ample evidence that lipophilic statins, such as SIM, are more efficient than hydrophilic statins in improving cardiac function and decreasing inflammation, minimizing their drawbacks is still needed^[^^10^^,^^11^^]^. 

Recently, with the advent of nanoparticles, various drug delivery systems have emerged to elevate drug potency without increasing the cytotoxicity risk. In this regard, studies have shown that the smaller size of nanocarriers is associated with higher biodistribution, lower toxicity, and an increased half-life of drugs^[^^12^^,^^13^^]^. For instance, the nanoniosome, a novel vesicular nanoparticle, is based on non-ionic surfactants and cholesterol^[^^14^^]^. Therefore, owing to the non-ionic property in an aqueous phase, this nanoparticle is considered a perfect option for improving the poor aqueous solubility and stability of lipophilic agents such as SIM^[^^15^^,^^16^^]^. For the evaluation of SIMloaded nanoniosomes function, comparing the impact of SIM with that of SIMloaded nanoniosomes on cell viability is important.

One of the most common cell death pathways during reperfusion injury is necroptosis, which has been recognized as a caspase-independent cell death pathway^[^^17^^]^. Inhibiting necroptosis through necrostatin-1 administration reduces in vitro and in vivo cardiac IR/I^[^^18^^,^^19^^]^. Furthermore, RIPKs are a class of serine/threonine protein kinases, play a vital role in inflammation, immune responses, and death-inducing processes. Among these kinases, RIPK1 is the most crucial necroptosis marker, which initiates cell death once phosphorylated^[^^20^^]^. Moreover, the expression level of *HMGB1* increases in the ischemic myocardium, which is associated with ‎an impaired cardiopulmonary system. This ubiquitous nuclear protein acts as an important mediator of inflammatory disorders including IR/I. There is evidence of a negative correlation between *HMGB-1* expression levels and structural and functional ‎cardio-vascular impairment^[^^21^^-^^23^^]^. 

Nrf2 is a pivotal transcription factor that controls the expression level of several antioxidant elements, which strengthen the antioxidant defense system of cells in either physiological or pathological conditions such as heart failure and myocardial infarction^[^^24^^-^^26^^]^. It has clearly been understood that once excessive ROS is produced, an increased expression level of *Nrf2* enables cardiomyocytes to neutralize oxidative stress, resulting in maintaining oxidant-antioxidant balance^[^^27^^-^^29^^]^. 

In the present study, we selected embryonic rat myocardium-derived cells (H9c2) to induce an in vitro OGD/R injury, which mimics the I/RI in cardiomyocytes^[^^25^^,^^30^^,^^31^^]^. For the first time, this study investigates the impact of SIMloaded nanoniosomes on an in vitro model of OGD/R disease, with focusing on oxidative stress, necroptosis, and inflammatory pathways.

## MATERIALS AND METHODS

**Preparation and characterization of nanoniosomes**

The preparation and characterization of nano-niosomes, such as drug encapsulation and release, were explained in our prior investigation^[^^15^^]^. In summary, nanonoisomes were prepared using a film hydration method with surface-active agents. Following 5 minutes of stirring at 60 °C (150 rpm), polyethylene glycol, Span-60, and cholesterol were dissolved in absolute ethanol. Next, the solvent was vaporized using a rotary evaporator (Heidolph, Germany), and SIM was then added to the nanoniosomes. SIMloaded nanoniosomes were placed in a carbon-coated copper grid at 80 kV. TEM and dynamic light scattering (He-Ne laser) were used to measure the size and shape of the nanoparticles^[^^17^^]^. The size of nanoniosomes with or without SIM was measured, and their morphology was characterized by a zeta laser (wavelength 632.8 nm; Malvern, Zetasizer Nano ZSE, Worcestershire, UK). Fourier transform infrared spectrum (400–4000 cm^-1^; Nicolet, Avatar 360 FT-IR spectrometer, USA) was used to evaluate the entrapment efficacy of the agent. 

**Cell culture**

H9c2 cells, purchased from the Pasteur Cell Bank (Tehran, Iran), were cultured in 25-cm^2^ flasks in a DMEM medium containing 10% FBS and 100 U/mL of penicillin/streptomycin (Gibco, USA). After incubating in a humidified 5% CO_2_ atmosphere at 37 °C for 48 h, the cells were passaged every 3-4 days.

**Treatment groups and model induction**

Cells were seeded based on five groups: (1) control, (2) OGD/R, (3) OGD/R receiving nanoniosomes, (4) OGD/R receiving SIM, and (5) OGD/R receiving SIMloaded nanoniosomes. Cells in the OGD/R group were deprived of oxygen and nutrients for four hours to induce an in vitro I/RI model, meaning that serum-free and glucose-free DMEM were used instead of the culture medium. Moreover, the cells were incubated in hypoxic conditions (95% N_2_ and 5% CO_2_) at 37 °C for four hours. Reperfusion was established by exposing H9c2 cells to DMEM containing 10% FBS under standard conditions, including 95% air and 5% CO_2_ at 37 °C, which lasted for 48 h^[^^14^^]^. SIM, nanoniosome, or SIM-loaded nanoniosomes were added to the cells once the reperfusion initiated. 

**H9c2** **cell viability**

The effect of OGD/R, SIM, nanoniosome, and SIMloaded nanoniosomes on the viability of H9c2 cells was evaluated. In this regard, H9c2 cells were seeded in 96-well plates at a density of 5 × 10^3^ cells/well for 48 h, and in case of the control group, the cells were kept intact in the incubator. After two days of reperfusion, the cell viability was assessed using the MTT test (Sigma-Aldrich, USA). Finally, the absorbance was measured by a microplate reader (Model 550; Bio-Rad, USA) at 570 nm. The cell viability was presented as the percentage of the control, and the test was repeated three times.

**TNF-α and IL-1β determination**

To determine inflammatory markers, we assessed TNF-α and IL-1β levels, 48 hours after reperfusion. At first, 40 µl of supernatant from all groups was transferred to a plate of 96 wells, and then 10 µl of antibodies TNF-α (catalog no. RK00027, Abclonal, USA) and IL-1β (catalog no. RK00001, ELISA ZellBio kit, Germany) was added. Subsequently, 50 µl of streptavidin-horseradish peroxidase solution was added to the wells and incubated at 37 °C for 1 hour. Then the wells were washed four times for 30 seconds. Next, 50 µl of each chromogen A and B solution was added to the wells. After incubation in the dark at 37 °C for 10 min, the stopping solution was added to all the wells, and the absorbance was measured with a microplate reader at 450 nm. The ELISA test was repeated three times, and each specimen was tested in triplicate.

**Gene expression measurement by real-time PCR**

The *Nrf2* and *HMGB1* gene expressions were assessed using real-time PCR. In brief, real-time PCR was carried out to synthesize the first-strand complementary DNA using a reverse transcriptase kit (Qiagen, Germany) by extracting total RNA from H9c2 cells. Afterwards, a reverse transcription reaction was performed by applying the following forward and reverse primers: 5´-AACAACACTGCTGCGGATG-3´ and 5´-TCTCAA GTACAATCCCCTCACA-3´ for *HMGB1*, 5´-CACGG TGGAGTTCAATGACT-3´and 5´-GAAGAATGTGT TGGCTGTGC-3´ for *Nrf2*, and 5´-TGACAGGATGCA GAAGGAGA-3´ and 5´-TAGAGCCACCAATCCA CACA-3´ for *β-actin*, respectively. Each PCR reaction was carried out using PCR master mix and SYBER Green in ABI Step One (Applied Biosystems, Sequences Detection Systems, Foster City, CA, USA, for both). The PCR reactions were conducted as follows: 95 °C for 5 min, 40 cycles of 95 °C for 10 s, 60 °C for 30 s, and 72 °C for 30 s. β-actin was applied as a control. Data were analyzed by the 2^-ΔΔCT^ method.

**Western blotting analysis**

Western blotting test was performed to assess the necrosis markers, RIPK1 and ROCK1. In summary, H9c2 cells were harvested, washed twice with PBS and minced in a lysis buffer containing protease and phosphatase inhibitors cocktail. Next, the cell lysate was centrifuged at 161 ×g at 4 °C for 20 min, and the supernatant was collected. The protein quantification was evaluated by the Bradford test. Afterwards, 50 μg of the whole protein was run on a 12% SDS-PAGE gel and separated with electrophoresis based on their molecular weight. Proteins were then transferred to PVDF membranes (Immobilon®-FL PVDF membrane, pore size 0.45 μm, Sigma-Aldrich), which were sealed with 5% fat-free milk at 25 °C for one hour. The membranes were incubated with anti-RIPK1‎ and anti-ROCK1 primary antibodies at 4 °C overnight and exposed to diluted secondary antibodies at 25 °C for one hour. Chemiluminescence was applied to detect protein bands, and Alpha Innotech FluorChem FC2 Imaging System was employed to analyze band densities. *β-actin* was applied as the housekeeping gene. 

**Statistical analysis**

The results of this research were presented as the mean ± SEM. Differences were compared by a one-way ANOVA test, followed by Tukey’s post hoc test using Graph Pad Prism software. Statistical significance was considered at *p* < 0.05.

## RESULTS

**Nanonoisome characteristics**

**Fig. 1:**
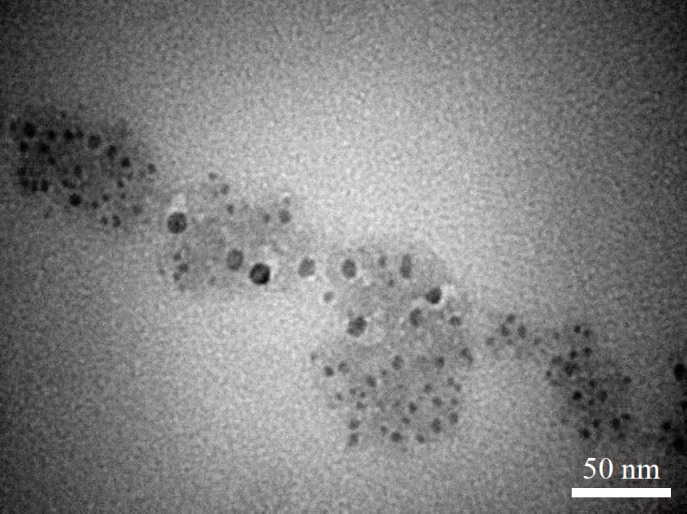
TEM image of nanoniosome

The TEM image showed the spherical shape of nanoniosomes with a two-layer structure. Their average size was about 30-60 nm (Fig. 1).

**Fig. 2:**
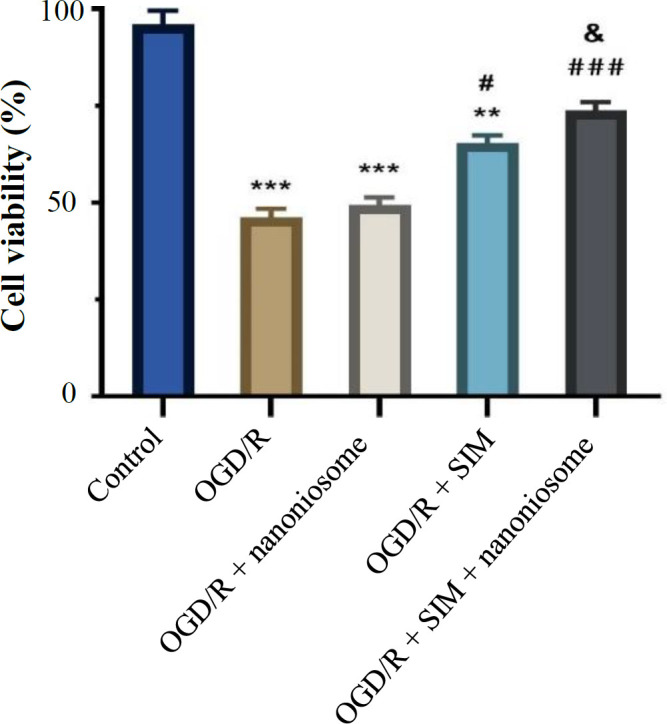
Impact of SIM and SIM-loaded nanoniosomes on the cell viability 48 h after the induction of OGD/R injury.‎ The results are presented as mean ± SEM (^***^p < 0.001 and ^**^p < 0.01, vs. control; ^#^*p* < 0.05 and ^###^*p* < 0.001 vs. OGD/R; ^&^*p* < 0.05 vs. SIM)

**Cell viability assay**

Cell viability significantly reduced in the OGD/R group in comparison to the control (*p* < 0.001). Based on the MTT results shown in Figure 2, the cell viability increased in the SIM group compared to the OGD/R group and also in the SIM-loaded nanoniosomes compared to the SIM group (*p* < 0.05). As shown in Figure S1, 48 hours after reperfusion, the cells’ morphology was assessed in different groups using light microscopy. 

**Cytokine assessment**

**Fig. 3:**
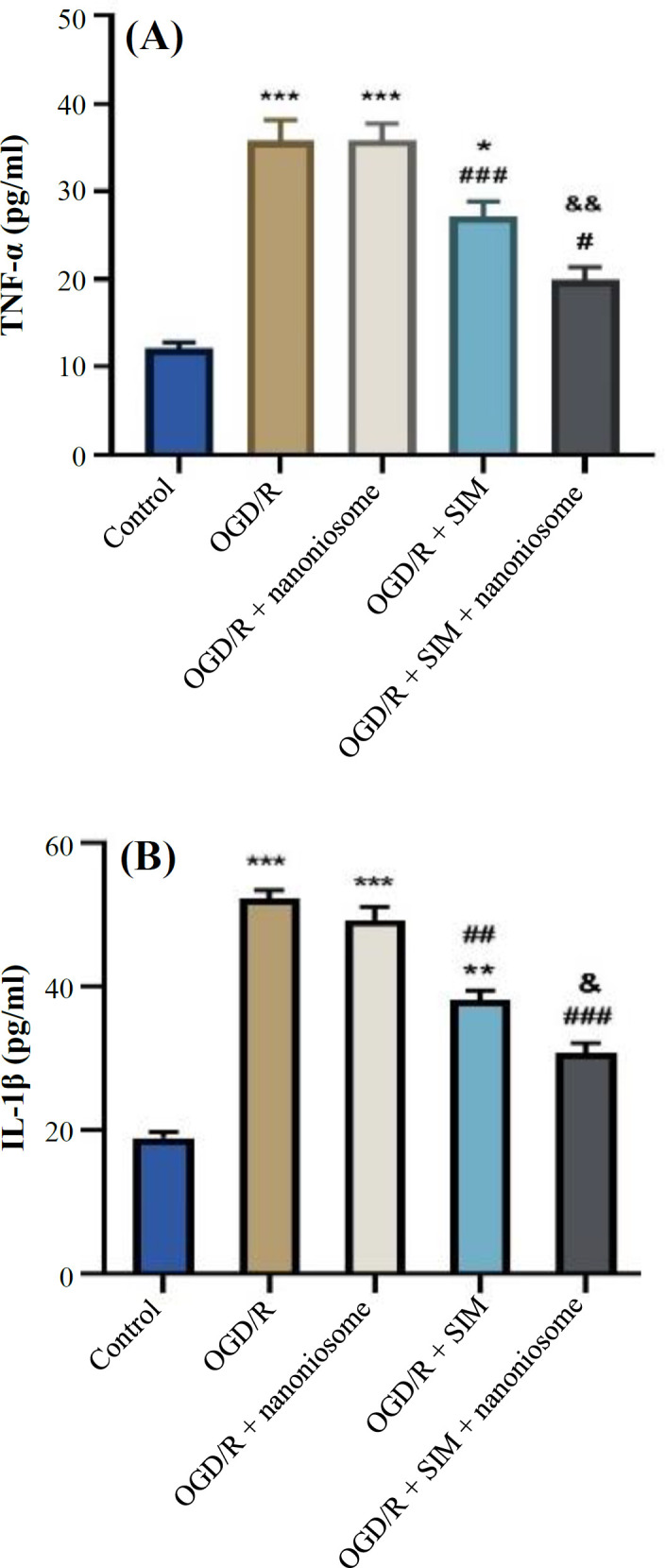
Impact of SIM and SIM-loaded nanoniosomes on the (A) TNF-α and (B) IL-1β inflammatory cytokines, 48 hours after the induction of OGD/R injury using ELISA test. The results are presented as mean ± SEM (^***^p < 0.001, ^**^p < 0.01, and ^*^p < 0.5 vs. control; ^###^*p* < 0.001, ^##^*p *< 0.01, and ^#^*p *< 0.05 vs. OGD/R; ^&&^*p* < 0.01 and ^&^*p *< 0.05 vs. SIM)

The ELISA test showed more TNF-α and IL1-β production in the OGD/R group compared to the control *p* < 0.001). The results also showed that SIM-loaded nanoniosomes was more potent than SIM alone in decreasing the inflammatory response (Fig. 3).

**Gene expression level assay**

**Fig. 4:**
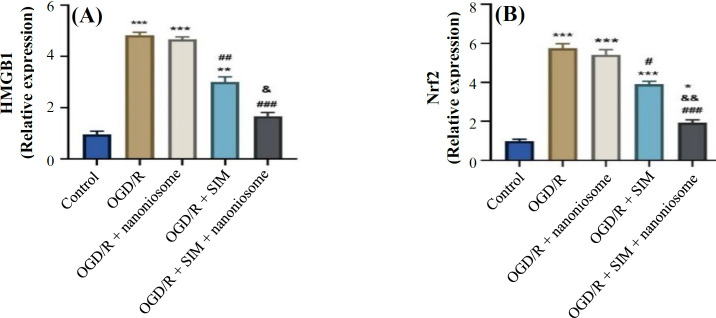
Effects of SIM and SIM-loaded nanoniosomes on the expression of (A) *HMGB1* and (B) *Nrf2*, 48 hours after the induction of OGD/R injury using real-time PCR. The results are presented as means ± SEM (^***^p < 0.001, ^**^p < 0.01, and ^*^p < 0.5 vs. control; ^###^*p *< 0.001, ^##^*p* < 0.01, and ^#^*p* < 0.05 vs. OGD/R; ^&&^*p* < 0.01 and ^&^*p* < 0.05 vs. SIM)

As depicted in Figure 4, the OGD/R group had much higher levels of *HMGB1* expression than the control group (*p* < 0.001). *HMGB1* can initiate the inflammatory cascade, which leads to the cell damage. The results showed increased *HMGB1* expression level in the OGD/R + SIM group compared to the control (*p* < 0.001). There was no statistical difference in the expression level of *HMGB1* in the OGD/R + nanoniosome compared to the OGD/R group. In addition, Nrf2 decreased in the cells treated with SIM in comparison to untreated hypoxia (*p* < 0.05). This reduction was higher in the nanoniosome-treated group than the SIM-treated group (*p* < 0.05). The *HMGB1* (*p* < 0.05) and *Nrf2* (*p *< 0.01) expressions were lower in the OGD/R + SIM + nanoniosome group than the OGD/R + SIM group.

**Evaluation of necrosis factors**

**Fig. 5:**
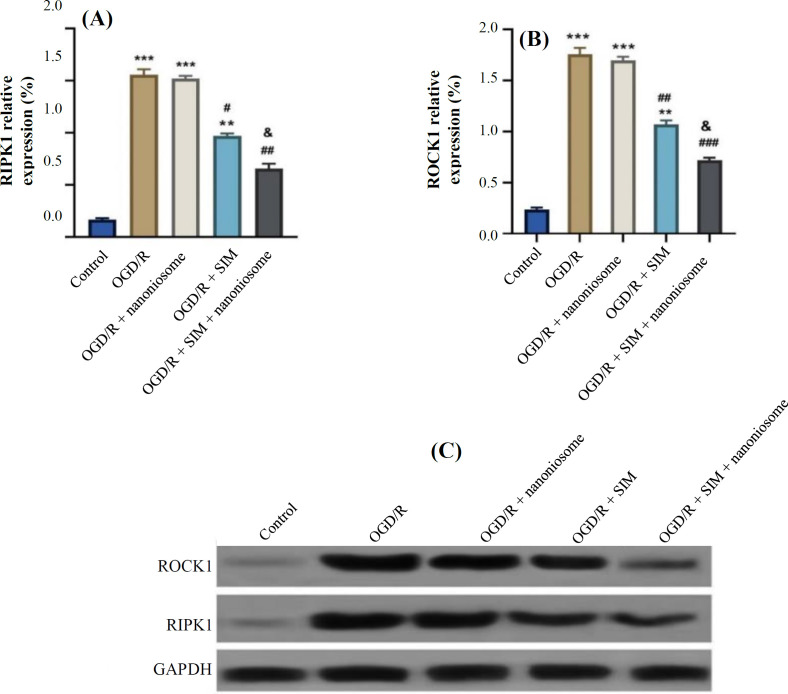
Impact of SIM and SIM-loaded nanoniosomes on the expression level of (A) RIPK1 and (B) ROCK1 proteins 48 h after the induction of OGD/R injury using Western blotting analysis. (C) The expression of RIPK1, ROCK1, and β-actin proteins. The results are presented as means ± SEM (^***^p < 0.001 and ^**^p < 0.01 vs. control; ^###^*p* < 0.001, ^##^*p* < 0.01, and ^#^*p* < 0.05 vs. OGD/R; ^&^*p* < 0.05 vs. SIM)

Western blotting analysis revealed that RIPK1‎ was significantly upregulated in the OGD/R group in comparison to the control (*p* < 0.05). The data also indicated that ROCK1 was downregulated in the SIM group in comparison to the OGD/R one. This reduction was higher in the OGD/R + SIM + nanoniosome group than that of the OGD/R + SIM group (*p* < 0.05; Fig. 5). 

## DISCUSSION

Myocardial ischemia is a severe stress scenario that causes oxidative stress and massive innate immune and inflammatory responses, eventually resulting in extensive cardiomyocyte loss^[^^32^^,^^33^^]^. In cultured cardiac myocytes, glucose deprivation causes oxidative stress, as evidenced by the overproduction of ROS^[^^31^^]^. In addition, SIM could mitigate cell death and IR-mediated myocardial injury; however, its low bioavailability makes it an inappropriate agent^[^^15^^]^. On the other hand, studies have shown that the controlled release of bioactive molecules has significant therapeutic advantage^[^^12^^,^^13^^,^^34^^]^.

Nanonisome is a vesicle mainly formed by cholesterol and non-ionic surfactants and have a bilayer structure in common with liposomes; however, its nature has made it more stable than liposomes^[^^35^^]^. Herein, we loaded SIM into nanoniosomes to increase their solubility and efficacy to treat OGD/R-induced H9c2 cell injury. Moreover, lipid nanoparticles have been absorbed easily into the membrane of targeted cells^[^^36^^]^. 

In this investigation, the size of nanocarriers was 30-60 nm. We found that OGD/R primarily induced necroptosis in H9c2 cells, with *RIPK1*‎ and *ROCK1* underexpression after OGD/R induction. Treatment with 1 μM of SIM decreased *RIPK1*‎ and *ROCK1* expression levels in hypoxic H9c2 cells compared to the control. Additionally, these markers significantly decreased in the OGD/R+ SIM + nanoniosome group, as compared to the OGD/R + SIM group. The level of inflammatory factors, TNF-α and IL-1β, increased after 48-hour reperfusion, while treating the cells with SIM + nanoniosome significantly reduced these factors, compared to the OGD/R+SIM group. Our study showed that SIM protects the cardiomyocytes against OGD/R-induced disorder by inhibiting inflammation and cell death, and loading SIM into nanoniosomes markedly increased its therapeutic effects.

In parallel with our report, Salem et al. have shown that SIM-loaded niosomal gel has more appropriate percutaneous delivery than SIM^[^^37^^]^. In our prior study, the effects of nanoniosomes on enhancing cardiac function and obstructing the necroptosis pathway were more effective in the SIM + nanoniosome group than in the SIM group^[^^15^^]^. Findings of the current study indicate that the use of nanoniosomes as a significant drug delivery system can improve the stability, bioavailability, and curative effectiveness of SIM when it is utilized to treat myocardial I/RI^[^^15^^]^. In agreement with our results, Durak et al. studied the application of liposomal drugs in treating ocular disorders and introduced nanoniosomes as promising nanocarriers in treating ocular diseases^[^^34^^]^. Abou-Taleb et al. also showed that nefopam-loaded niosomes have ability to increase the penetration of nefopam through nasal mucosa increased and ameliorate its relative bioavailability compared to nefopam oral solution^[^^38^^]^. Furthermore, Kanaani et al. reported that liposomes increase the cytotoxic impact of cisplatin by 1.5 times in comparison to the standard drug and stated that nanoniosomes are suitable carriers for the delivery of cisplatin to breast tumor cells^[^^39^^]^. 

## CONCLUSION

Our study demonstrates that nanoniosomes could safely be applied as a drug delivery system to counterbalance the disadvantages of SIM, as well as improve the poor aqueous solubility and effectiveness of this lipophilic statin.

## DECLARATIONS

### Acknowledgments

No artificial intelligence was used in this study.

### Ethical approval

 This study was approved by the Research Ethics Committee of Faculty of Pharmacy and Pharmaceutical Sciences, Islamic Azad University of Medical Sciences, Tehran, Iran (ethical code: IR.IAU.PS.REC.1400.191). 

### Consent to participate

 Not applicable.

### Consent for publication

All authors reviewed the results and approved the final version of the manuscript.

### Authors’ contributions

MN: designed the study; MMM: wrote the article; MN and MMM: performed the experiments and statistical analysis.

### Data availability

All relevant data can be found within the manuscript. 

### Competing interests

The authors declare that they have no competing interests. 

### Funding

 This research received no specific grant from any funding agency in the public, commercial, or not-for-profit sectors.

### Supplementary information

The online version contains supplementary material.
